# Concurrent superficial epidermolytic ichthyosis and generalized pustular psoriasis - Report of a case, review of the literature, and a proposed pathophysiologic link

**DOI:** 10.1016/j.jdcr.2025.05.006

**Published:** 2025-05-30

**Authors:** Jing Yi Han, Isabelle A. Vallerand, Ahmed Shah, Michelle M. Schneider, Lynne H. Robertson

**Affiliations:** a Cumming School of Medicine, University of Calgary, Calgary, Alberta, Canada; b Division of Dermatology, University of Calgary, Calgary, Alberta, Canada; c Department of Pathology and Laboratory Medicine, University of Calgary, Calgary, Alberta, Canada; d Department of Pathology, Duke University, Durham, North Carolina

**Keywords:** ichthyosis, pustular psoriasis, superficial epidermolytic ichthyosis

## Introduction

Superficial epidermolytic ichthyosis (SEI), also known as ichthyosis bullosa of Siemens is a rare, autosomal dominant, nonsyndromic subtype of congenital ichthyosis caused by mutations in the keratin 2e gene.^1^ Generalized pustular psoriasis (GPP) is an autoinflammatory disease characterized by sterile pustulosis with or without systemic symptoms. These conditions have recently been found to share common immunologic profiles.^2^ Herein, we present a patient with SEI and concurrent generalized annular pustular psoriasis. We review the literature on the coexistence of recurrent pustular eruptions and congenital ichthyoses and explore a potential pathophysiologic link between these 2 rare conditions.

## Case report

A 36-year-old Caucasian male presented for evaluation of a widespread pruritic pustular eruption. It began on the left forearm and spread over 3 weeks to involve both upper and lower extremities with sparing of the trunk, face, hands, and feet. There were no associated constitutional symptoms and no improvement had occurred with a 10-day course of cephalexin followed by a 5-day course of prednisone 50 mg trialed by another physician prior to presentation at our clinic. The patient reported similar but less severe episodes occurring on a monthly basis over the previous 10 years.

His past medical history was significant for hypertension and dyslipidemia managed respectively with bisoprolol and rosuvastatin. He also gave a history of widespread erythema at birth, episodic spontaneous blistering of the skin in childhood and severe generalized xerosis and thickened skin on the extensors beginning in adulthood. The patient had one unaffected sibling, but there was a family history of similar skin problems occurring in his mother and maternal grandfather. There was no family history of psoriasis.

**Fig 1:**
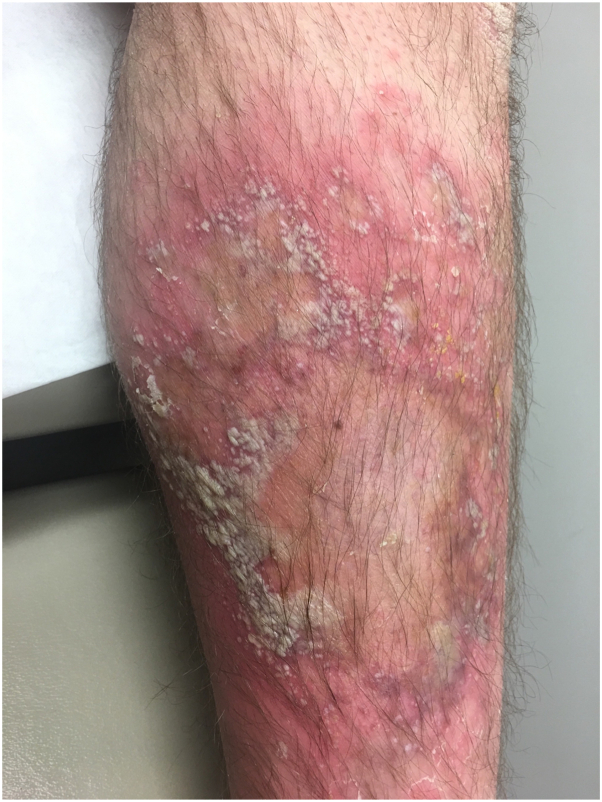
Ill-defined and annular erythematous plaques with overlying pustules, lakes of pus, and crusting on the inner arms.

**Fig 2:**
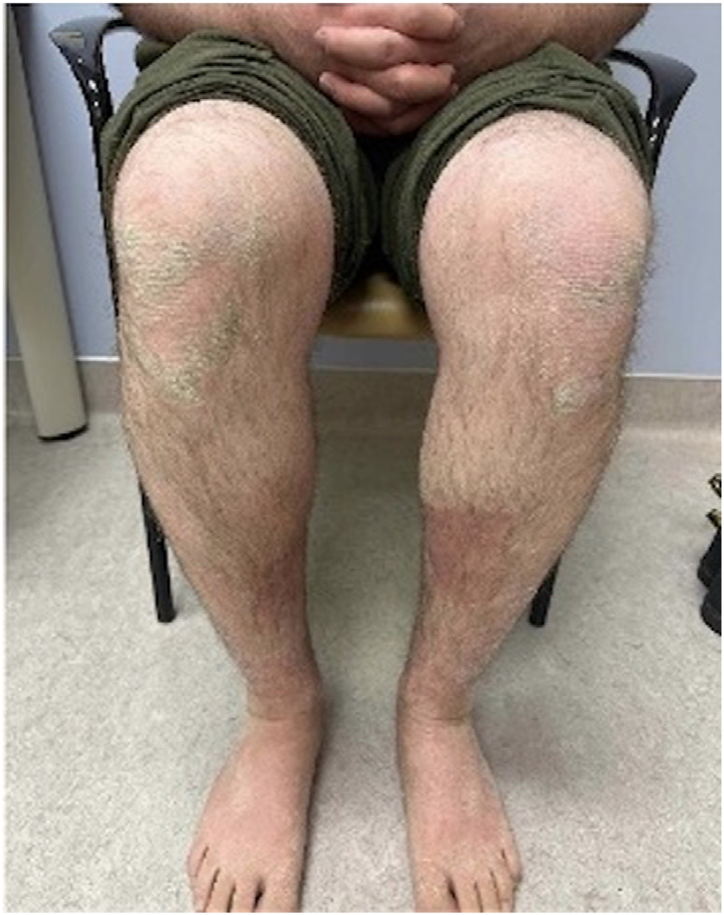
Hyperkeratotic plaques on the knees and ichthyosiform scaling on the legs.

Examination revealed numerous pinpoint pustules occurring on a background of erythema. In some areas, these coalesced into lakes of pus and were arranged in an annular configuration (Fig 1). There were numerous collarettes of scale noted. Examination of the scalp, nails and mucous membranes was normal. There was mild generalized xerosis, ill-defined hyperkeratotic plaques involving the knees and ichthyosiform scaling involving the lower legs (Fig 2).

**Fig 3:**
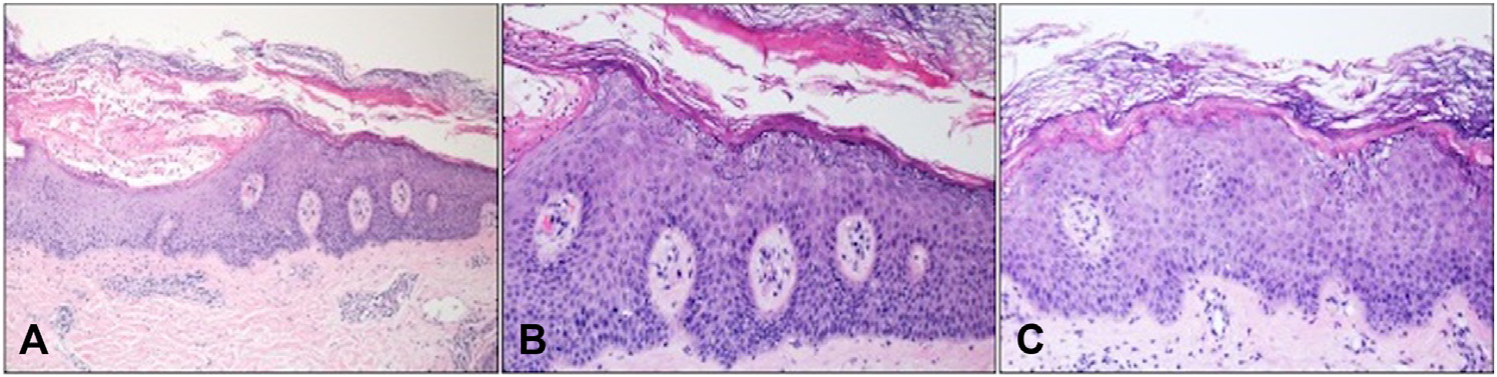
Representative images of right forearm biopsy specimen. **A,** Skin showing prominent subcorneal pustule on a background of acanthosis and hyperorthokeratosis (H&E, ×100). **B,** Skin adjacent to the pustule showing hyperorthokeratosis with a prominent granular layer consisting of large irregularly shaped keratohyaline granules. (H&E, ×200).**C,** Background skin showing hyperorthokeratosis, subtle superficial vacuolization of the keratinocytes in the granular layer and purple irregular clumps of keratohyaline granules (H&E, ×200).

A biopsy of the right forearm revealed a subcorneal pustule occurring in association with an acanthotic hyperorthokeratotic epidermis with a prominent granular layer containing large, irregularly shaped keratohyaline granules. These background changes were suggestive of an underlying disorder of keratinization, namely a keratinopathic ichthyosis. Direct immunofluorescence showed no significant uptake for IgG, IgA, IgM, or fibrin with non-specific and focal granular uptake of C3 in the basement membrane (Fig 3). Tissue stains and culture for bacteria, mycobacteria and fungi were negative. The recurrent nature of the pustulation, lack of an implicated drug, nonspecific direct immunofluorescence findings and negative cultures favoured a diagnosis of generalized pustular psoriasis occurring in association with a keratinopathic ichthyosis. Specific keratinopathic ichthyoses considered in the differential diagnosis included SEI, epidermolytic ichthyosis and annular epidermolytic ichthyosis. The mild nature of the ichthyosis, and absence of keratoderma and psoriasiform scaling of the recurrent annular erythematous plaques favored a diagnosis of SEI. Genetic testing on a blood sample identified a pathogenic variant of the KRT2 gene, specifically c.1459G>A (p.Glu487Lys), confirming a diagnosis of superficial epidermolytic ichthyosis. Mutations in pustular psoriasis-associated genes IL36RN and CARD14 were not identified. Treatment with acitretin 25 mg daily resulted in the resolution of the pustular episodes within 1 week and significant improvement in the patient's ichthyosis at 1 month.

## Discussion

SEI is a congenital keratinopathic ichthyosis caused by mutations in the keratin 2e gene protein with an estimated worldwide prevalence of 1:500,000.^3^ It is uniquely characterized by superficial peeling or “molting” described as the Mauserung phenomenon and has variable phenotypic presentations, including the presence or absence of erythroderma at birth, blistering of the skin, and lichenified hyperkeratosis mainly confined to flexural areas.^3^

Pustular psoriasis comprises 1% to 2% of all psoriasis subtypes and is clinically characterized by localized or generalized eruptions of superficial neutrophil-rich sterile pustules on an erythematous base. Generalized pustular psoriasis (GPP) is a systemic autoinflammatory disorder which has been linked to activation of the innate immune system and overactivation of IL-36 cytokine pathways. Phenotypic variants of GPP include an acute widespread pustular eruption (Von Zumbusch variant), a generalized pustular eruption occurring in pregnancy (impetigo herpetiformis), and a generalized annular subtype which manifests as erythematous circinate plaques studded with pustules.^4^ Historically therapeutic interventions for GPP have included oral retinoids, methotrexate and cyclosporin. More recently, biologics that target the IL-36 immune axis or specifically the IL-36 receptor are favored for acute severe disease.

The coexistence of GPP and ichthyosis has previously been reported. A review of the English literature identified 15 cases of pustular eruptions occurring in association with various forms of congenital ichthyoses, including bullous ichthyosiform erythroderma/epidermolytic ichthyosis, non-bullous ichthyosiform erythroderma/lamellar ichthyosis, ichthyosis vulgaris, and unspecified ichthyosiform dermatitis.^5^, ^6^, ^7^, ^8^, ^9^, ^10^, ^11^, ^12^, ^13^, ^14^, ^15^ Among these, there were 7 reports of generalized or localized pustular psoriasis occurring in the setting of SEI.^6^^,^^7^^,^^9^^,^^13^^,^^15^ Of the 5 male patients, 3 had onset of GPP in the teenage years; one had type 1 mosaic SEI caused by a somatic mutation in the KRT2 gene and onset of GPP in childhood, another had uncharacterized recurrent pustular eruptions starting in childhood. Two of the patients were female who had uncharacterized pustular eruptions beginning in infancy or adulthood. One adult male was successfully treated with etretinate 10-20 mg od and an infant male received transient improvement with systemic corticosteroids. Therapeutic intervention for the remaining 5 cases was not mentioned.

Several of these reports question whether the pustular eruptions represented pustular psoriasis or were a manifestation of the ichthyosis itself. However, more recent studies that demonstrate the presence of psoriasis-like immune dysregulation in congenital ichthyoses lend support for the coexistence of these 2 rare diseases.^4^

Several orphan ichthyoses including congenital ichthyosiform erythroderma, epidermolytic ichthyosis, lamellar ichthyosis, and Netherton syndrome, have been shown to have an IL-17/IL-23 and IL-36 dominant immune profile in skin and blood similar to psoriasis. Increased mRNA and protein expression of the IL-36 receptor and its cytokines IL-36α, IL-36β, and IL-36γ significantly correlate with ichthyosis severity and trans epidermal water loss, providing support to their role in the Th17 axis in the ichthyoses.^3^ Given IL-36α, β and γ are all agonists of the IL-36 receptor, they may play a pathogenic role in the occurrence of pustular psoriasis in patients with ichthyosis. We hypothesize that the episodic nature of the pustular eruptions may correlate with worsening ichthyosis disease severity and upregulation of these cytokines. The rare association of pustular psoriasis and ichthyosis suggests that this phenomenon may be exclusive to those ichthyosis patients who have a genetic predisposition to psoriasis and a baseline increase in IL-36 signaling.^11^

In summary, advances in the immune profiling of inflammatory diseases such as pustular psoriasis and ichthyoses provide insights into the pathophysiologic mechanisms underlying the coexistence of these rare disorders. Further, biologics that target shared cytokine signalling abnormalities are a promising therapeutic option in their concomitant management.

## Conflicts of interest

None disclosed.
